# Reactivation of mutant p53 in esophageal squamous cell carcinoma by isothiocyanate inhibits tumor growth

**DOI:** 10.3389/fphar.2023.1141420

**Published:** 2023-04-24

**Authors:** Lulu Guan, Yalan Yang, Yao Lu, Yu Chen, Xi Luo, Dao Xin, Xiangrui Meng, Zhengzheng Shan, Guozhong Jiang, Feng Wang

**Affiliations:** ^1^ Department of Oncology, The First Affiliated Hospital of Zhengzhou University, Zhengzhou, China; ^2^ Department of Pathology, The First Affiliated Hospital of Zhengzhou University, Zhengzhou, China

**Keywords:** esophageal squamous cell carcinoma, mutant p53, p53, phenethyl isothiocyanate, reactivation

## Abstract

p53 mutations are prevalent in human cancers; approximately half of patients with esophageal cancer present these mutations. Mutant p53 (mutp53) exerts oncogenic functions that promote malignant tumor progression, invasion, metastasis, and drug resistance, resulting in poor prognosis. Some small molecules have been shown to mitigate the oncogenic function of mutp53 by restoring its wild-type activity. Although these molecules have been evaluated in clinical trials, none have been successfully used in the clinic. Here, we investigated the antitumor effects of phenethyl isothiocyanate (PEITC) in p53-mutant esophageal squamous cell carcinoma (ESCC) and elucidated its mechanism to identify new therapeutic strategies. We observed that p53^R248Q^ is a DNA contact mutation and a structural mutation and that PEITC can restore the activity of p53^R248Q^ *in vitro* and *in vivo*, further clarifying the antitumor activity of PEITC in cancers with different types of p53 mutations. PEITC can inhibit ESCC growth, induce apoptosis, and arrest cell cycle progression and has a preferential selectivity for ESCC with p53 mutations. Mechanistic studies showed that PEITC induced apoptosis and arrested cells at G2/M transition in cells expressing the p53^R248Q^ mutant by restoring the wild-type conformation and transactivation function of p53; these effects were concentration dependent. Furthermore, PEITC inhibited the growth of subcutaneous xenografts *in vivo* and restored p53 mutant activity in xenografts. According to these findings, PEITC has antitumor effects, with its ability to restore p53^R248Q^ activity being a key molecular event responsible for these effects.

## 1 Introduction

World-wide, esophageal cancer is one of the most common cancers (Sung et al., 2021). Esophageal squamous cell carcinoma (ESCC) is the most frequent histological subtype (Yang et al., 2018). Despite significant advances in ESCC treatment, its 5-year overall survival rate is as low as 13% due to the high rates of metastasis and recurrence (Lagergren et al., 2017; Li et al., 2018; Zhang C. et al., 2019). For the development of novel targeted cancer therapies, understanding the molecular mechanisms underlying malignant tumorigenesis and progression is crucial (Lagergren et al., 2017; Turner et al., 2018). However, to date, there are still relatively few studies on esophageal cancer, and the main signaling pathways and molecular mechanisms underlying its development have not been fully elucidated (Shi et al., 2019). Thus, in order to develop new therapeutic modalities, we must investigate the molecular mechanisms underlying esophageal carcinogenesis and development.

p53 is the most commonly mutated gene in human tumors (Zhang et al., 2020). In the p53 gene, the majority of mutations are missense mutations, which fall into two categories: DNA contact mutations and structural mutations (Freed-Pastor and Prives, 2012; Yue et al., 2017). R248 is one of six hotspot residues that is frequently mutated in p53 (Muller and Vousden, 2014). Most studies classify R248 as a DNA contact mutation, whereas a few reports consider it a DNA contact mutation and structural mutation. p53 mutations lead to the inactivation of the tumor suppressor function of wild-type p53 (wtp53) and the concurrent gain of a range of oncogenic functions (Kastan and Berkovich, 2007). Missense mutations in p53 result in inactivation of its antitumor transcriptional response, and mutant p53 (mutp53) exerts oncogenic functions by affecting molecular regulation, altering metabolism, and interfering with many other pathways (Muller and Vousden, 2014; Shetzer et al., 2016; Mantovani et al., 2017; Pfister and Prives, 2017; Singh et al., 2017; Schulz-Heddergott and Moll, 2018). At least 50% of patients with esophageal cancer have p53 gene mutations, and the mutation rate is higher in patients who are active smokers and consume alcohol (Olivier et al., 2010; Szymanska et al., 2010; Agrawal et al., 2012), with the highest frequency of mutations occurring at R248 (6.4%, 280/4354), followed by R175 (5.6%, 244/4354) (http://www.p53.fr/). Therefore, it is paramount to target the p53 gene for treating esophageal cancer and to find new small-molecule compounds that restore the function of mutp53, which may lay the foundation for establishing targeted therapy for patients with ESCC expressing mutp53.

Isothiocyanates (ITCs) are a class of sulfur-containing organic compounds widely found in cruciferous plants. Phenethyl isothiocyanate (PEITC) is a representative member. PEITC has been studied in various phases of clinical trials (https://www.clinicaltrials.gov/). There has been strong evidence that ITCs are highly effective in the prevention and treatment of tumors and have high clinical value, in which the reactivation of mutp53 is crucial (Aggarwal et al., 2016; Zhang et al., 2016; Liu et al., 2018; Aggarwal et al., 2019; Wang et al., 2020); however, the specific mechanism by which ITCs restore the wild-type activity of mutp53 in ESCC has not been elucidated. Therefore, the relationship between ITCs and mutp53 in ESCC needs to be investigated. Herein, we focused on the p53^R248^ mutant and PEITC and on investigating the mechanism of action by which PEITC influences ESCC expressing mutp53.

## 2 Materials and methods

### 2.1 Cell culture

EC109, KYSE150 and 293T cells were obtained from The Cell Bank of Type Culture Collection of The Chinese Academy of Sciences. KYSE150, EC109 and 293T cells were cultured at 37°C in a humidified environment with 5% CO_2_ in medium containing 10% fetal bovine serum (FBS; Invigentech) and 1% Penicillin-Streptomycin Liquid (Solarbio, China), with the difference that the former two used RPMI-1640 (Sigma-Aldrich, Missouri, United States) and the latter used DMED (Sigma-Aldrich, Missouri, United States).

### 2.2 Cell viability assay and proliferation assay

A total of 2 × 10^3^ cells/well were plated in 96-well plates (Corning, NY, United States of America) and incubated overnight. Every 24 h, a total of 10 μL CCK-8 solution (Dojindo Molecular Technologies, Inc.) was added to cells, which were further incubated for 2 h. Absorbance was measured at 450 nm using a microplate reader to determine cell proliferation.

### 2.3 Colony formation assay

In six-well plates, 300 (KYSE150) or 500 (EC109) cells were seeded per well, after adherent, cells were treated with DMSO or PEITC for 2 weeks at 37°C with 5% CO_2_. PEITC was dissolved in DMSO and further diluted with culture medium before use. After incubation, the cells were fixed with 4% paraformaldehyde for 20 min, then stained for 15 min with 0.1% crystal violet (Solarbio, China). Finally, pictures were taken, and ImagesJ was used to count how many colonies were present in the photos.

### 2.4 EdU incorporation assay

A kFluor647 Click-iT EdU Imaging Assay Kit (KeyGEN BioTECH, China) was used for the EdU incorporation assay. A total of 4 × 10^4^ cells/well were cultured for 36 h in RPMI1640 medium (supplemented with 10% FBS) in 12-well plates. After adherent, cells were treated with DMSO or PEITC for 24 h. Following incubation with the EdU solution (50 mM) for 4 h at 37°C, the cells were fixed in 4% paraformaldehyde for 20 min at room temperature. Then, the cells were permeabilized for 17 min with 0.5% Triton X-100 and reacted for 30 min with Click-iT reaction cocktail (500 µL). The nuclei were dyed for 20 min at ambient temperature with Hoechst 33,342 and observed with a fluorescence Olympus microscope (Olympus, Japan).

### 2.5 Flow cytometric analysis

With the Annexin V-FITC/propidium iodide (PI) apoptosis detection kit (KeyGEN BioTECH, China), apoptosis was detected. A total of 1.2 × 10^6^ cells were seeded in 10 cm cell culture dishes. A 12-h incubation with DMSO (Solarbio, China) or PEITC (Sigma-Aldrich, Missouri, United States) was carried out after overnight incubation. Following harvest and washing with PBS, the cells were suspended in 500 µL binding buffer containing 5 µL of annexin V-FITC and 5 µL of PI for 15 min at room temperature out of the light. Apoptosis was analyzed by an ACEA NOVOCYte3130 flow cytometer (Agilent Technologies, California, United States).

For cell cycle analysis, a total of 1.2 × 10^6^ cells were seeded in 10 cm cell culture dishes. A 12-h incubation with DMSO or PEITC was carried out after overnight incubation. After harvesting, for fixing, the cells were rinsed with sterile PBS, then centrifuged (2000rmp for 5min, 4°C), washed twice, counted, and resuspended in 70% cold ethanol for 2 h at −20°C. After that, the cells were washed and centrifuged twice, resuspended in 500 µL of PI/RNase staining solution (KeyGEN BioTECH, China) and incubated in darkness for 30 min. The samples were analyzed using an ACEA NOVOCYte3130 flow cytometer (Agilent Technologies, California, United States).

### 2.6 RNA extraction and reverse transcription-quantitative PCR (RT-qPCR)

Cells for RT-qPCR were treated with DMSO or PEITC for 24 h. Cell RNA was extracted using RNAiso Plus (TaKaRa, Japan). A NanoDrop 1,000 (Thermo Scientific, MA, United States) was used to measure the RNA concentration and quality. RNA was reverse transcribed to cDNA using a PrimeScript™ RT reagent Kit with gDNA Eraser (Perfect Real Time) (TaKaRa, Japan) in a 20 μL total volume. Gene expression was measured using UltraSYBR Mixture (Low ROX) (CWBIO, China) and analyzed using QuantStudio™ Design & Analysis Software (Life Technologies, Carlsbad, CA, United States). Sangon Biotech synthesized the primers for qPCR, and their sequences are presented in Supplementary Table S1. Normalization was performed using GAPDH mRNA as a reference. The relative expression was calculated using the 2^−ΔΔCT^ method.

### 2.7 Western blotting

Cells for Western blotting were treated with DMSO or PEITC for 24 h. Total cell proteins were extracted using RIPA lysis buffer containing protease and phosphatase inhibitors. A BCA protein assay kit (Solarbio, China) was used to detect the protein concentrations. 30 μg total proteins were loaded on an SDS-gel, separated using SDS-PAGE, and then transferred to PVDF membranes (Merck Millipore, MA, United States of America). Membranes were subsequently blocked in 5% skimmed milk at ambient temperature for 2 h, followed by incubation with anti-GAPDH (Proteintech, #10494-1-AP), anti-p53 (abcam, #ab131442), anti-cleaved caspase 3 (CST, #9664), anti-PAb240 (Merck Millipore, #OP29L), anti-PAb1620 (Merck Millipore, #OP33), anti-p21 (CST, #2947), anti-Bcl2 (Proteintech, #12789-1-AP), anti-BAX (CST, #14796), anti-PUMA (Proteintech, #55120-1-AP), anti-MDM2 (Proteintech, #27883-1-AP), anti-CyclinB1 (abcam, #ab32053) or anti-p73 (abcam, #ab215038) overnight at 4°C. The membranes were then rinsed in TBST for 20 min and incubated for 1 h with the secondary antibody (Affinity Biosciences, Cincinnati, OH, United States) at room temperature. Signals were detected using enhanced chemiluminescence reagent (Affinity Biosciences, Cincinnati, OH, United States) and imaged using an AI600 series imager (GE Healthcare, Carlsbad, CA, United States).

### 2.8 Lentivirus package and transfection

To knockdown p53 in cells, a GV112 vector containing p53 sh#1/sh#2/sh#3 sequences was constructed by GeneChem (Shanghai, China). As a negative control, an empty vector was used. The constructed plasmid was mixed proportionally with the psPAX2 packaging plasmid and pMD2. G envelope plasmid and transfected together into 293T cells to generate lentivirus. After 72 h of incubation at 37°C, lentiviruses overexpressing shp53 or shCon were collected and mixed with polybrene (10 mg/mL; Solarbio, China) and culture medium. A lentivirus infection was then carried out by adding the mixture to cells. Stably transfected cells were selected for by culturing transfected cells with 3 μg/μL or 1.5 μg/μL puromycin.

Firefly luciferase lentiviruses were constructed by GeneChem (Shanghai, China). Firefly luciferase lentivirus was mixed with HistransG A virus infection reagent and medium and added to the cells for lentiviral infection, and transfection efficiency was detected under a live imager by adding D-fluorophore potassium salt after 3 days.

### 2.9 Cell immunofluorescence staining

An appropriate amount of cell suspension was seeded onto polylysine-treated coverslips in 12-well plates, and cells were treated with DMSO or PEITC for 24 h after adherent. Then, 4% paraformaldehyde was applied to fix the cells after they were washed with PBS. Three rinses with PBS were followed by 16 min of permeabilization with 0.5% Triton X-100. Permeabilization was subsequently terminated with 0.1% BSA and incubated with anti-p21 (CST, #2947), anti-PAb240 (Merck Millipore, #OP29L) or anti-PAb1620 (Merck Millipore, #OP33) primary antibody at 4°C overnight. At room temperature, cells were incubated for 1 h with fluorescent secondary antibody. The nuclei of the cells were stained with DAPI for 5 minutes. Finally, the coverslips were analyzed with a positive fluorescence microscope (OLYMPUS, Japan).

### 2.10 Coimmunoprecipitation

Coimmunoprecipitation (CoIP) was performed using the Minute™ Total Protein Extraction Kit for Animal Cultured Cells/Tissues (Invent Biotechnologies, United States). Cells for CoIP were treated with DMSO or PEITC for 24h. SN-002 buffer was used to lyse the cells, and a BCA protein assay kit (Solarbio, China) was used to measure protein concentrations. We incubated lysates with antibodies overnight at 4°C before incubating them with protein A agarose for 2 hours. We washed beads three times with 1× PBS and boiled them with SDS-PAGE Sample Loading Buffer (5×, with DTT) (Leagene Biotecnology, China). For the analysis of protein interactions between immunoprecipitated samples, immunoblotting was performed. The anti-p53 (Proteintech, #10442-1-AP), anti-PAb240 (Merck Millipore, #OP29L), anti-PAb1620 (Merck Millipore, #OP33) and anti-p73 (abcam, #ab215038) were used.

### 2.11 Chromatin immunoprecipitation (ChIP)

Our experiments were conducted using the SimpleChIP^®^ Enzymatic Chromatin IP Kit (Magnetic Beads) (Cell Signaling Technology, MA, United States). Anti-p53 (Proteintech, #10442-1-AP) was used. SimpleChIP^®^ universal qPCR Master Mix (CST, #88989) was used for qPCR. The primers for ChIP-qPCR were as follows: 5′- ACA​TGT​TGA​GCT​CTG​GCA​TAG​A-3′ (forward) and 5′-ggg​gtc​ttt​aga​ggt​ctc​ctg​t-3′ (reverse). Relative enrichment was calculated by normalizing to IgG immunoprecipitation. In the end, agarose gel electrophoresis was used to analyze the PCR products.

### 2.12 Tumor xenograft in nude mice

Forty 5-week-old female athymic nu/nu BALB/c mice were bought from Charles River in Beijing (Vital River). We randomly divided the mice into four groups (n = 10 per group). KYSE150, shp53-KYSE150, EC109 and shp53-EC109 cells (2 × 10^6^) suspended in PBS were injected subcutaneously into the right mid-posterior axilla of 6-week-old female nude mice. After 5 days, mice inoculated with different cells were randomly divided into two groups (n = 5 per group) and given DMSO (<1/10,000 in PBS) or PEITC (10 μmol/100 μL/day) by gavage for 21 days or until the mice were killed due to excessive tumor load. At every 3 days, the weights of the mice and the volume of the tumors were measured. Xenograft volumes were measured by caliper measurements of two perpendicular diameters and calculated individually with the following formula: volume = l× w^2^/2, where l represents the maximal length, and w represents the width. The xenograft samples were taken for histological examination (paraffin section) or snapped into liquid nitrogen to preserve them.

### 2.13 Hematoxylin and eosin (HE) staining and immunohistochemistry (IHC)

Mice tumor tissues were soaked in 4% paraformaldehyde at room temperature, fixed for 24 h, embedded in paraffin, then cut into slices of about 4 mm and mounted onto slides. For histopathological analysis, we stained the tissue with HE. p53 (abcam, #ab131442) or Ki67 (Proteintech, #27309-1-AP) antibodies were used to stain specimens for immunohistochemistry. In brief, we heated the sections at 60°C for 2 h, dewaxed them in xylene, and dehydrated them with gradient concentrations of alcohol. After microwave antigen retrieval of sections, endogenous peroxidase was blocked with hydrogen peroxide, followed by blocking with goat serum to block non-specific staining, and primary antibodies were then evenly added to sections and incubated overnight at 4°C. The sections were then incubated at 37°C for 10 min with a horseradish peroxidase-conjugated secondary antibody. Subsequently, with diaminobenzidine (DAB) solution and hematoxylin counterstained, the sections were visualized. Finally, dehydration was performed, and the sections were sealed with Permount TM Mounting medium.

### 2.14 Statistical analysis

All cell experiments were performed in triplicates. All statistical analyses in this study were performed with SPSS 22.0 software (SPSS, Chicago, United States) and GraphPad Prism software 8.0 (GraphPad Software, CA, United States). Quantitative data are presented as the means ± SD. Differences between groups were analyzed using a one-way ANOVA followed by a *post hoc* Tukey’s multiple comparisons test or an unpaired Student’s t-test. ^*^ *p* < 0.05 was judged as statistically significant.

## 3 Results

### 3.1 ESCC cells with p53 mutation are inhibited by PEITC

**FIGURE 1:**
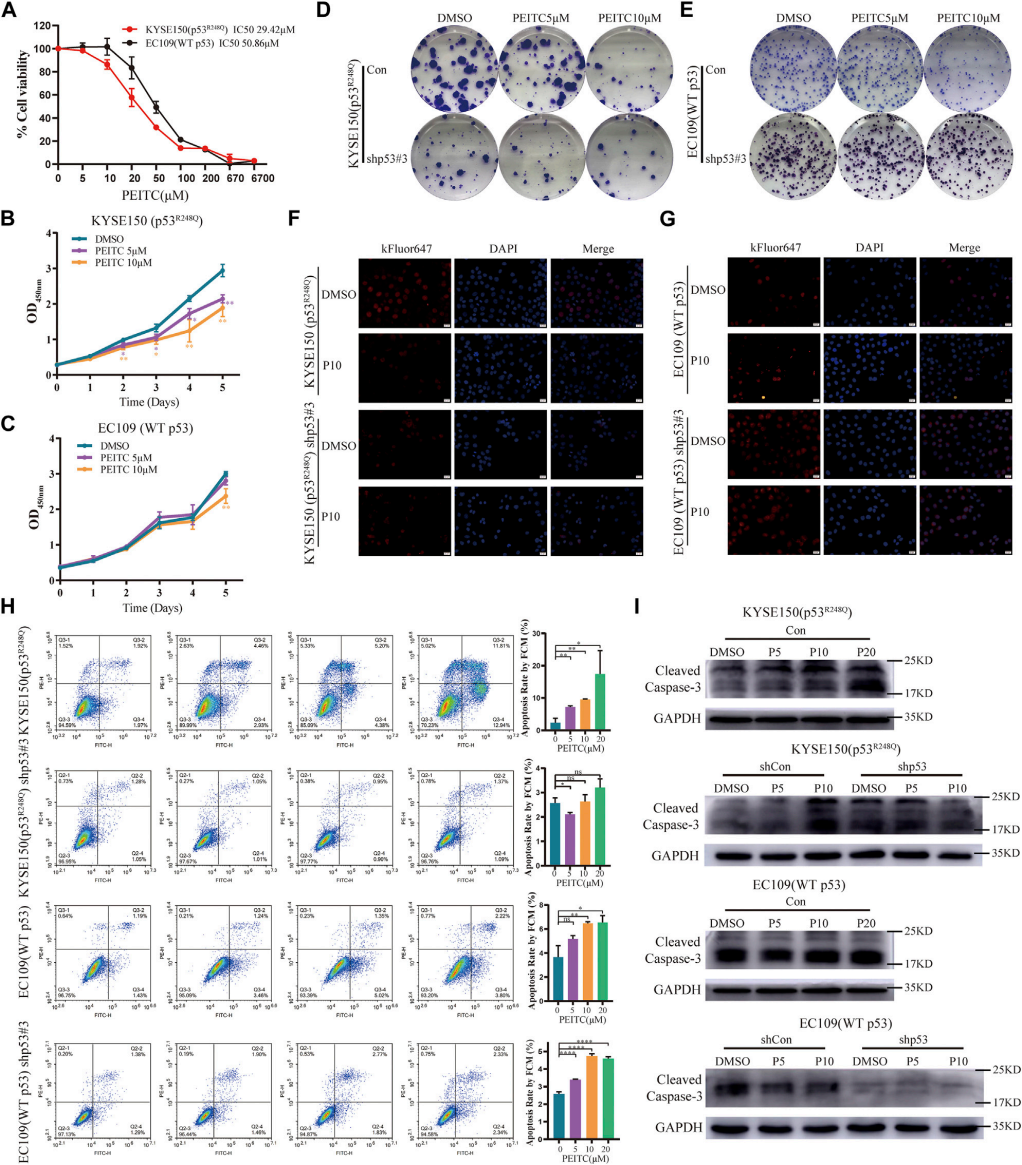
PEITC preferentially inhibits the proliferation of p53-mutant ESCC cells, and its effects are p53^R248Q^-dependent. **(A)** Cell viability in each group was measured by the CCK-8 assay, and the IC50 is indicated. Cells lines were treated with DMSO or PEITC for 2 days. **(B, C)** CCK-8 assay to detect the effect of DMSO or PEITC on the proliferation of cells. **(D–G)** Colony formation and EdU incorporation assays were used to analyze the proliferation of cells after treatment with DMSO or PEITC for 2 weeks or 24h. Scale bar = 20 μm. **(H)** Cell apoptosis was detected by flow cytometry. Cells were treated with DMSO or PEITC for 12h. **(I)** The protein expression levels of cleaved caspas3 and GAPDH were measured by Western blot analysis. *p < 0.05; **p < 0.01. P5, PEITC 5μM; P10, PEITC 10 μM. The cell experiment was repeated three times.

To examine the role of PEITC in the proliferation of ESCC cells with different p53 statuses, we treated the human ESCC cell lines KYSE150 (p53^R248Q^) (p53 gene mutation information from COSMIC database and Supplementary Figure S1) and EC109 (WT p53) with PEITC. EC109 and KYSE150 cells were inhibited to varying degrees by PEITC (Figure 1A). As compared with EC109 cells, PEITC significantly inhibited the proliferation of KYSE150 cells (Figures 1A–C). PEITC inhibited KYSE150 proliferation preferentially was also observed in clone formation and EdU incorporation assays. It was found that KYSE150 cells treated with PEITC had a significantly lower number of colony-forming and EdU-positive cells than EC109 cells (Figures 1D–G, Supplementary Figure S2). These results indicate that PEITC inhibits the proliferation of ESCC cells and that cells expressing the p53^R248Q^ mutation are more sensitive to its inhibition of growth.

### 3.2 PEITC inhibits proliferation and induces apoptosis in a p53^R248Q^-dependent manner

In order to investigate whether inhibition of ESCC cell proliferation by PEITC is mediated by restoring p53^R248Q^ activity, we transfected control shRNA (shCon) or p53-targeted shRNA (shp53) into ESCC cells. A more than 70% reduction in p53 mRNA and protein expression was observed in cells transfected with shp53 (Supplementary Figure S3). We discovered that the inhibition of shp53-KYSE150 cell growth mediated by PEITC was significantly weaker than that of shCon-KYSE150 cell growth (Supplementary Figure S4A). By contrast, EC109 cells transfected with shCon or shp53 did not exhibit significant differences in proliferation (Supplementary Figure S4B). Similar phenomena were observed in the clone formation and EdU incorporation assays (Figures 1D–G, Supplementary Figure S2). These results suggest that PEITC’s growth inhibitory effect appears to be at least partially dependent on p53^R248Q^.

Apoptosis was 1.5- to 3-fold higher in KYSE150 cells treated with PEITC than in EC109 cells treated with the same treatment (Figure 1H). It is important to note that DMSO or PEITC treatment of shp53-KYSE150 cells resulted in no significant differences in apoptosis (Figure 1H). Surprisingly, PEITC significantly increased apoptosis rates in shp53-EC109 cells compared to DMSO (Figure 1H). Furthermore, a concentration-dependent increase in cleaved caspase 3 expression was observed in KYSE150 cells treated with PEITC compared with DMSO, while shp53-KYSE150 cells did not show a significant increase in cleaved caspase 3 expression (Figure 1I). No PEITC-induced changes in cleaved caspase 3 expression were observed in either EC109 or shp53-EC109 cells (Figure 1I), indicating that p53^R248Q^ is partially responsible for PEITC-induced apoptosis.

### 3.3 PEITC induces conformational changes in p53^R248Q^ mutant proteins

**FIGURE 2:**
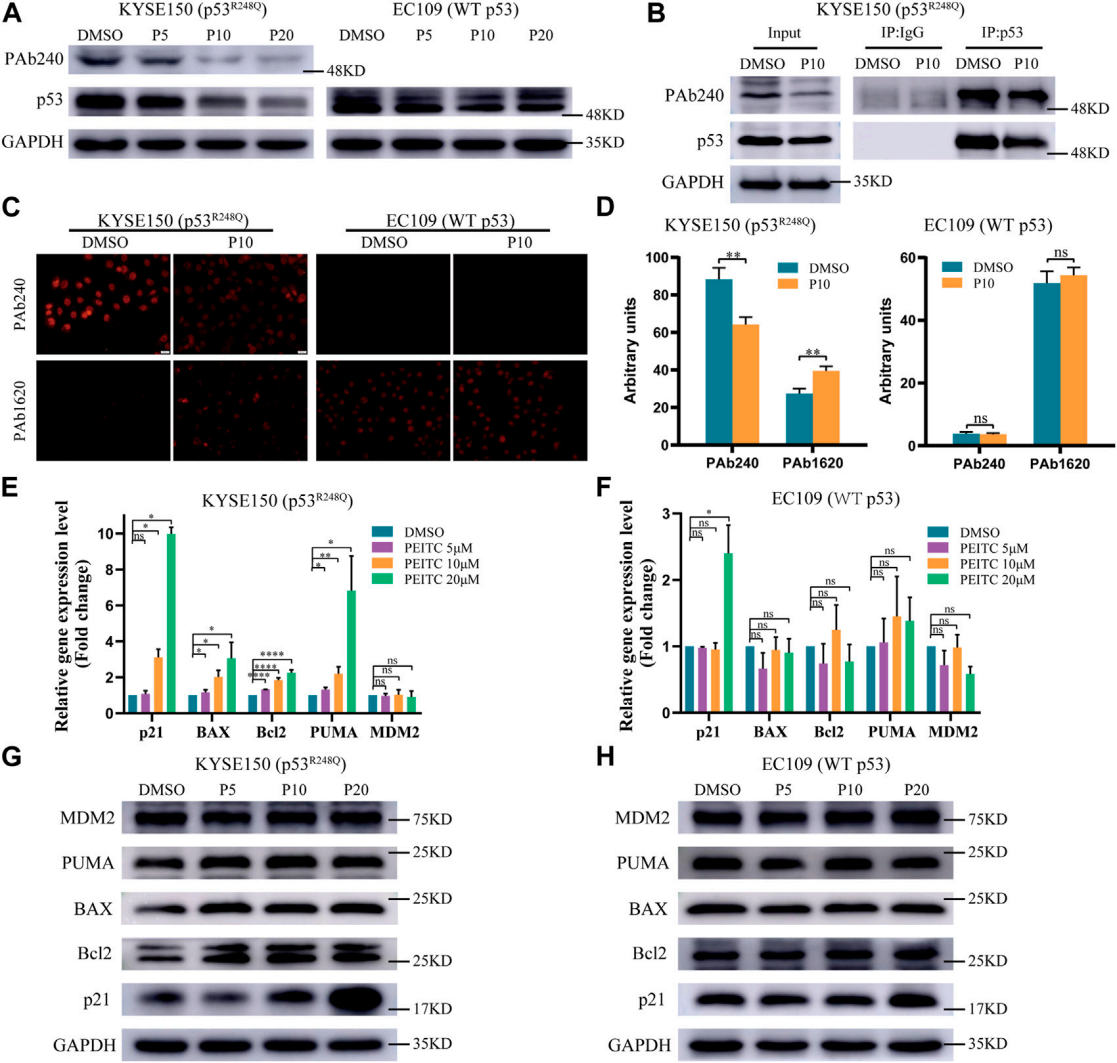
PEITC induces conformational changes in the p53^R248Q^ mutant protein and restores its transactivation function. **(A,G and H)** The expression levels of p53, PAb240, p21, Bcl2, BAX, PUMA, MDM2 and GAPDH proteins were measured by Western blot analysis. **(B)** Extracts of cells treated with DMSO or PEITC for 24h were subjected to p53 immunoprecipitation, and then to immunoblotting. **(C)** Immunofluorescence of the cells was performed using PAb240 and PAb1620 antibodies. **(D)** Quantification of PAb240 and PAb1620 staining shown in panel **(C)**. **(E, F)** RT-qPCR of p53-regulated genes in cells treated with DMSO or PEITC for 24 h *p < 0.05; **p < 0.01; ****p < 0.0001. P5, PEITC 5μM; P10, PEITC 10μM; P20, PEITC 20 μM. The cell experiment was repeated three times.

Considering PEITC inhibits cell growth and induces apoptosis in a mutp53-dependent manner, we speculated that PEITC restores the catalytic function of mutp53. Detection of p53 protein in tumor tissue can replace most of the detection of p53 mutations (Yue et al., 2017). Western blot analysis confirmed this phenomenon, with significantly higher p53 protein accumulation in KYSE150 cells than in EC109 cells (Supplementary Figure S1B). Compared with EC109 cells, KYSE150 cells had significantly reduced p53 protein expression levels after treatment with PEITC (Figure 2A), suggesting that mutp53 protein levels were low. Through the use of wtp53-specific antibody PAb1620 and mutp53-specific antibody PAb240, we investigated whether PEITC could restore the conformation of the p53^R248Q^ mutant. As compared with DMSO-treated cells, PEITC-treated cells showed a significant reduction in PAb240 binding in their lysates (Figures 2A,B). Upon treatment with PEITC, KYSE150 cells displayed an increase in PAb1620 antibody fluorescence, while PAb240 antibody fluorescence decreased (Figures 2C,D). These results show that PEITC can alter the spatial conformation of the p53 mutant protein to a more “WT-like” conformation.

### 3.4 PEITC restores the DNA-binding and transactivation functions of the p53^R248Q^ mutant protein

To determine the extent of the restored transduction function of p53 mutants by PEITC, we assessed the mRNA and protein expression levels of p53 regulatory genes in PEITC-treated KYSE150 cells. PEITC significantly increased the expression of the typical wtp53 targets p21, BAX and PUMA in p53-mutant KYSE150 cells, and the expression of Bcl2 was also increased, but it did not significantly affect MDM2 expression (Figures 2E,G). The expression levels of p53 target genes in EC109 cells treated with PEITC were not significantly altered (Figures 2F,H), suggesting that PEITC-mediated regulation of p53 target genes functions through p53^R248Q^. Consistent with the above results, compared with DMSO-treated KYSE150 cells, PEITC-treated cells exhibited significantly greater fluorescence intensity for p21 protein in immunofluorescence results, as for EC109 cells, no difference was observed (Supplementary Figure S5). p53 knockdown resulted in diminished p21 fluorescence intensity, and neither shp53-KYSE150 nor shp53-EC109 were significantly affected by PEITC for p21 expression (Supplementary Figure S5).

**FIGURE 3:**
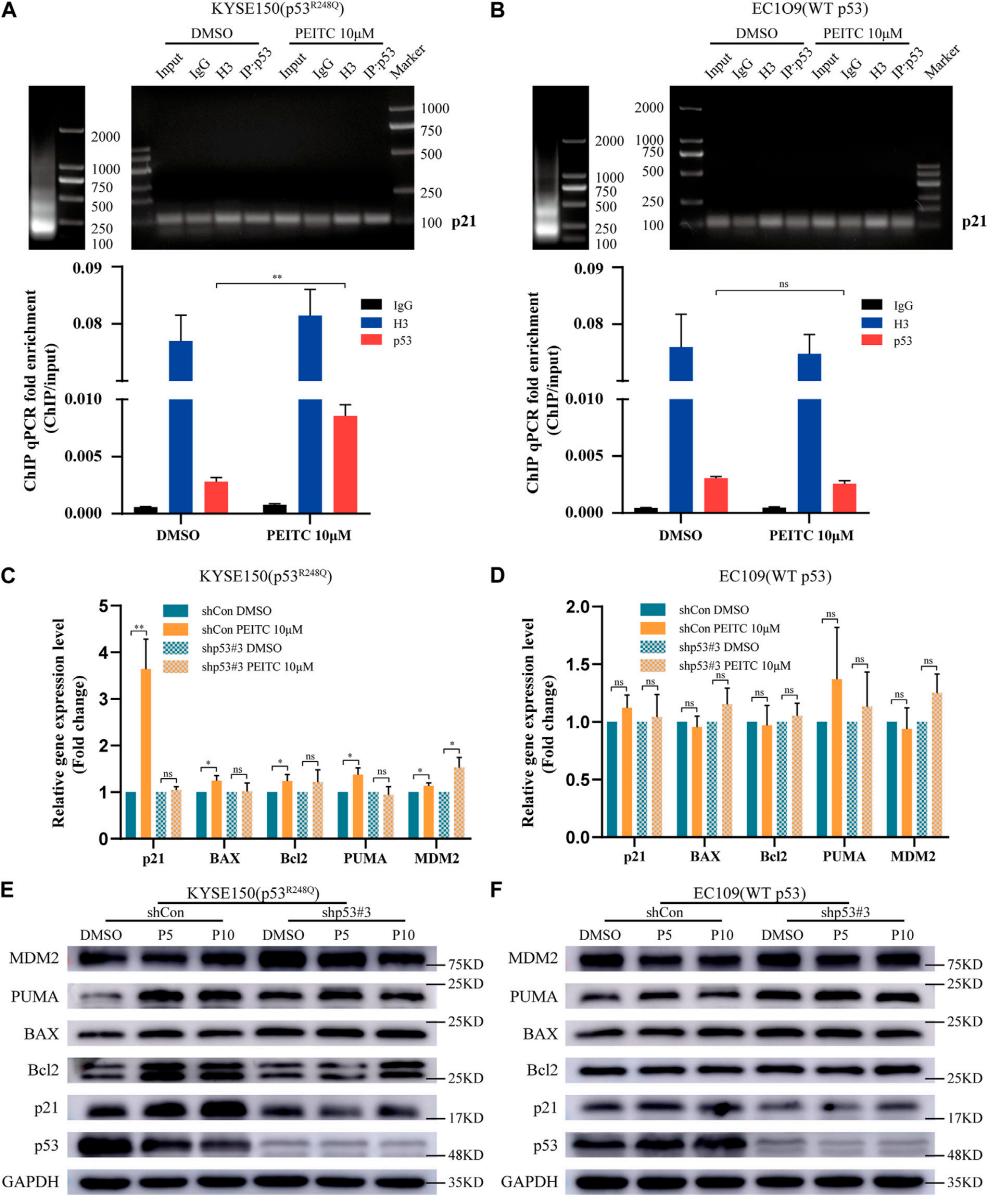
PEITC restores the DNA binding ability of the p53^R248Q^ mutant, and the restoration of the transactivational functions by PEITC is p53 mutant-dependent. **(A, B)** ChIP was performed to verify that PEITC restores the binding of the p53^R248Q^ mutant to the p21 promoter. Left, lanes showing the size of the ultrasonicated product. Right, lanes showing the p21 promoter detected by agarose gel electrophoresis. Below, statistical histogram. Input refers to the whole lysate of cells. **(C, D)** RT-qPCR of p53-regulated genes in cells treated with DMSO or PEITC for 24 h **(E, F)** The expression levels of p53, p21, Bcl2, BAX, PUMA, MDM2 and GAPDH proteins were measured by Western blot analysis. *p < 0.05; **p < 0.01. P5, PEITC 5μM; P10, PEITC 10 μM. The cell experiment was repeated three times.

Mutations in the p53 gene prevent it from binding to consensus DNA binding sequences, causing it to lose its tumor suppressor activity. Therefore, we investigated p21 using ChIP assay. Compared with DMSO, PEITC activated p21 expression to a greater extent in KYSE150 cells (Figure 3A), which was not observed in EC109 cells (Figure 3B), further demonstrating the restorative effect of PEITC on the transcriptional activity of p53^R248Q^. Based on these data, it can be concluded that PEITC induces a conformational change in p53^R248Q^ and restores its transactivation function.

Next, we assessed the effect of PEITC on shp53-KYSE150 cells. In RT-qPCR and Western blot analyses, it was found that in shCon-KYSE150 cells, PEITC increased the mRNA and protein expression levels of p21, BAX, PUMA and Bcl2 compared to those observed in response to DMSO treatment, while in shp53-KYSE150 cells, no significant change in the mRNA and protein expression levels of p21, BAX, PUMA and Bcl2 was observed after treatment with PEITC (Figures 3C,E). In addition, a significant difference was not observed in PEITC-induced expression levels of canonical p53 targets in shCon- or shp53-EC109 cells (Figures 3D,F). According to these findings, the PEITC-mediated restoration of transactivation functions in ESCC cells may be dependent on the presence of a p53 mutation.

### 3.5 PEITC induces cell cycle arrest in KYSE150 cells

**FIGURE 4:**
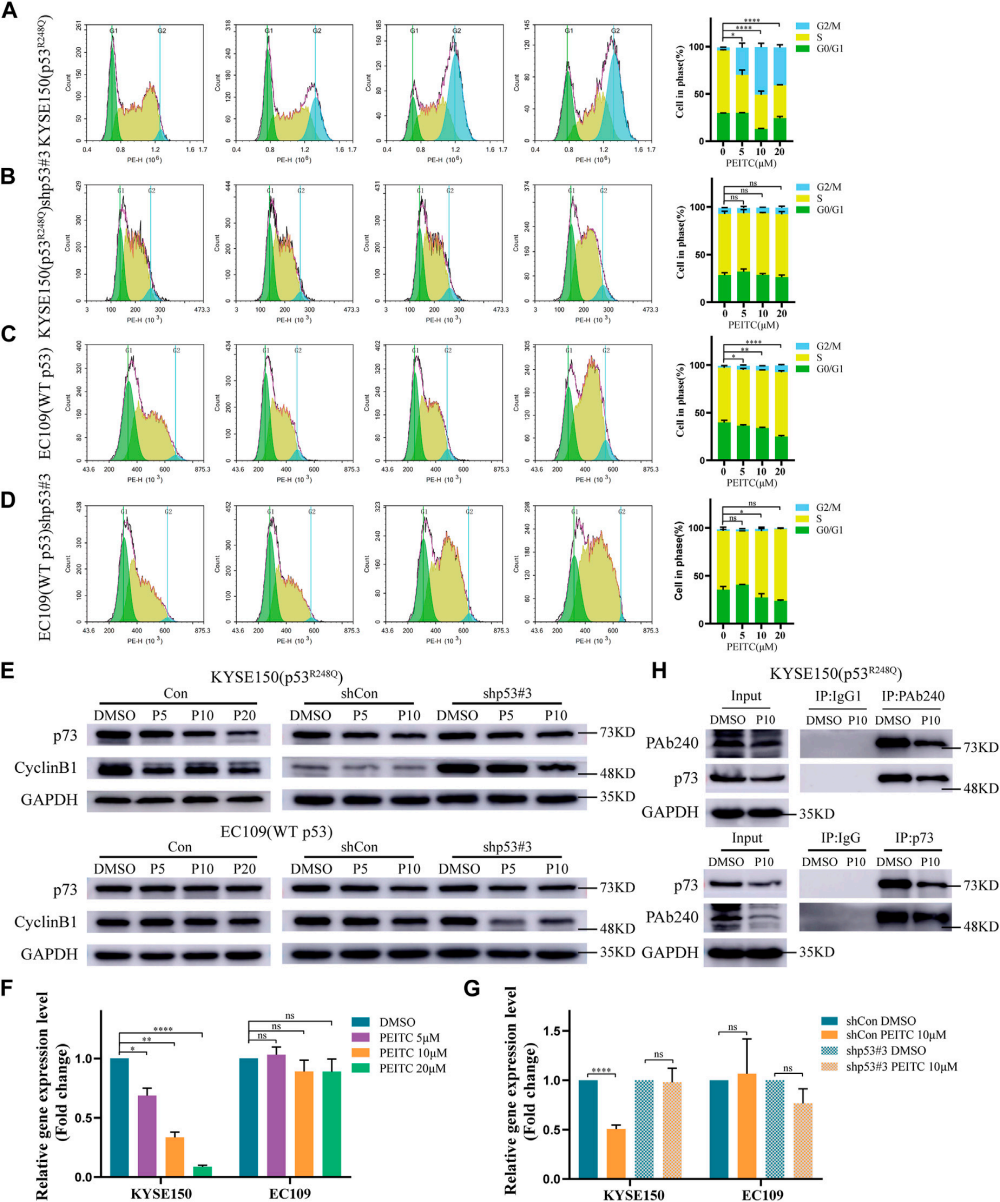
PEITC induces cell cycle arrest and the role of p73 in PEITC reactivation of p53^R248Q^. **(A–D)** The cell cycle was detected by flow cytometry. **(E)** The expression levels of p73, CyclinB1 and GAPDH proteins were measured by Western blot analysis. **(F, G)** mRNA expression level of p73. **(H)** CoIP assays confirming the interaction of the p53 mutant with p73 in KYSE150 cell lines. *p < 0.05; **p < 0.01; ****p < 0.0001. P5, PEITC 5μM; P10, PEITC 10μM; P20, PEITC 20 μM. The cell experiment was repeated three times.

p53 has a crucial role in regulating cell cycle progression and maintaining genetic stability (Jiang et al., 2018). We explored whether the reactivated p53^R248Q^ could regulate cell cycle progression. Compared to DMSO-treated cells, PEITC treated KYSE150 cells were dose-dependently arrested at G2/M transition; arrest at G2/M transition was also observed in PEITC treated EC109 cells, but the percentage was approximately one-tenth that of PEITC-treated KYSE150 cells (Figures 4A,C). No significant changes were observed in shp53-KYSE150 cells and shp53-EC109 cells (Figures 4B,D), indicating that PEITC-induced cell cycle arrest is partially dependent on p53^R248Q^. We then examined the CyclinB-cdc2 complex using Western blot analysis and found that CyclinB1 protein levels were significantly reduced in KYSE150 cells treated with PEITC compared to DMSO, whereas no significant changes were observed in EC109 cells (Figure 4E). Similarly, CyclinB1 protein levels were not significantly altered in shp53-KYSE150 and shp53-EC109 cells (Figure 4E). These findings further suggest that PEITC restores the transactivating function of p53^R248Q^.

### 3.6 The role of p73 in PEITC-mediated p53^R248Q^ activity restoration

As a member of the p53 family, p73 is an important tumor suppressor gene that has been reported to replace p53 to suppress tumor growth and drug resistance in tumors with dysregulated p53 function (Gomes et al., 2019). Therefore, we investigated the relationship between p73 and mutant p53 and found that PEITC significantly reduced p73 mRNA and protein expression in KYSE150 cells compared with DMSO-treated cells (Figures 4E,F). By contrast, in EC109, shp53-KYSE150 cells and shp53-EC109 cells, the p73 mRNA and protein expression levels in cells treated with PEITC were not significantly different from those treated with DMSO (Figures 4E–G). A mutation in p53 could lead to the inhibition of the tumor suppressor function of p73 when it binds to it. Our CoIP results indicated that the binding of mutantp53 to p73 was reduced in PEITC-treated KYSE150 cells compared with DMSO-treated cells (Figure 4H). It is suggested that PEITC may suppress the binding of mutant p53 to p73 to some extent and led to the conclusion that PEITC-mediated restoration of p53-mutant transactivation may negatively affect p73.

### 3.7 PEITC inhibits the growth of KYSE150 cell-derived xenografts and restores the activity of p53^R248Q^ *in vivo*

**FIGURE 5:**
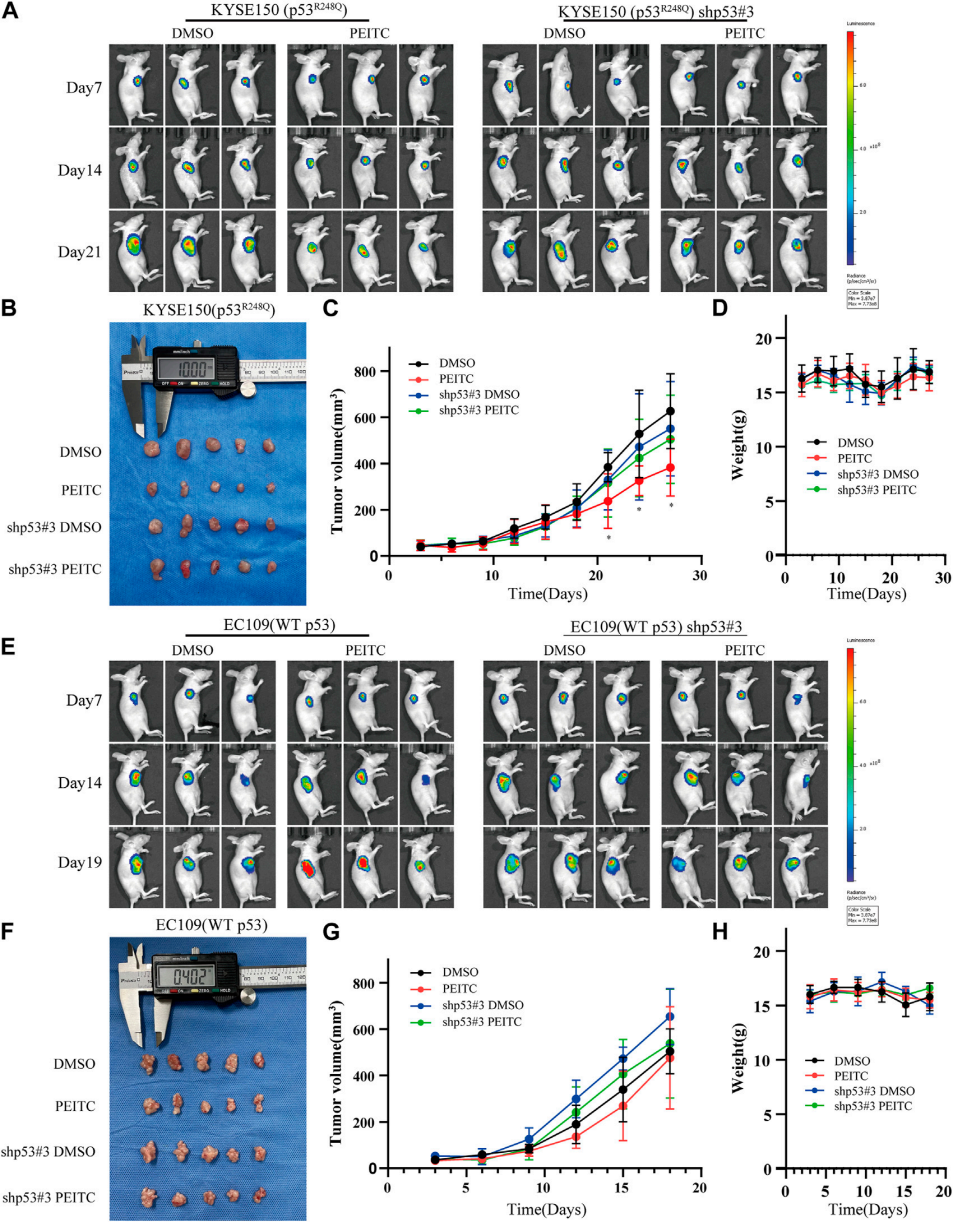
PEITC inhibits KYSE150 xenograft tumor growth. **(A, E)** Tumor growth measured by *in vivo* luciferase imaging of xenografts posttransplantation. **(B, F)** Tumor volumes of xenografts when the mice were killed. **(C, G)** Changes in tumor volume over time. **(D, H)** Changes in body weight of mice with time. *p < 0.05.

**FIGURE 6:**
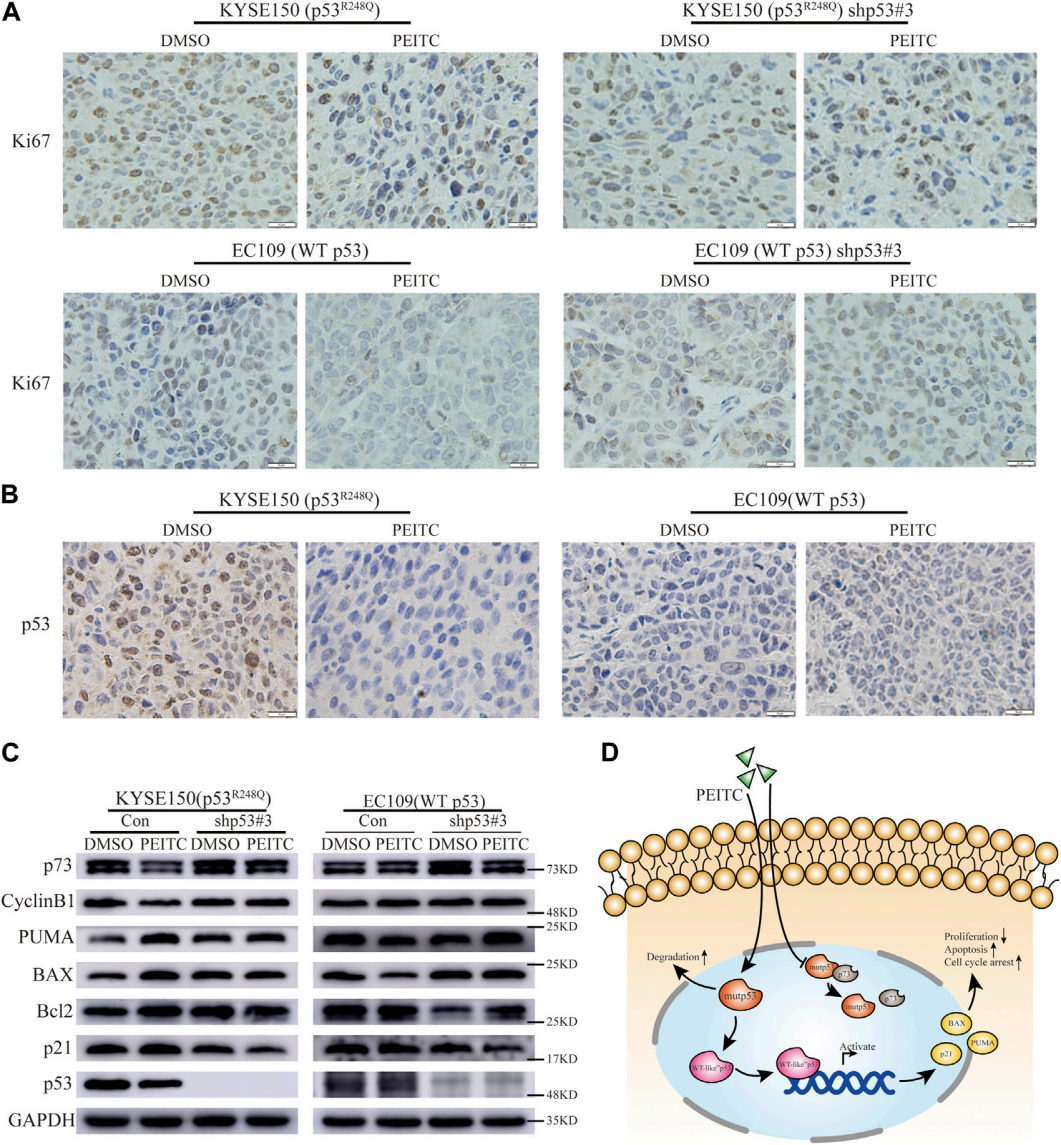
PEITC reactivates p53^R248Q^ *in vivo*. **(A)** IHC images of Ki67 (Scale bar = 10 μm). **(B)** IHC images of p53 (Scale bar = 10 μm). **(C)** Western blot showing p53, p21, Bcl2, BAX, PUMA, CyclinB1, p73 and GAPDH protein expression in tumor tissues. **(D)** Possible mechanisms of action of PEITC in p53 mutant ESCC.

Gavage administration was continued for 21 days or until the mice were sacrificed due to the excessive size of their tumors. A statistically significant reduction in tumor growth was demonstrated in the mice transplanted with KYSE150 cells and treated with PEITC gavage compared that of mice treated with DMSO (Figures 5A–C). Additionally, PEITC-treated animals had significantly fewer Ki67-stained cells in their tumors (Figure 6A). In contrast, similar phenomena were not observed in the nude mouse models of the remaining three cell types (Figure 5; Figure 6A). These results provide conclusive evidence that PEITC exerts antitumor activity against ESCC KYSE150 xenografts.

Consistent with *in vitro* experiments, IHC and Western blot analysis revealed that in the KYSE150 nude mouse transplantation tumor model, a significantly smaller percentage of p53-positive staining cells and significantly lower p53 protein expression levels were present in the tumors in the PEITC group compared to the DMSO control group (Figures 6B,C). However, the EC109 nude mouse transplantation tumor model did not show this phenomenon (Figures 6B,C). The results suggest that PEITC is able to eliminate the mutp53 protein *in vivo*.

Then, we explored whether the p53 mutant could be reactivate by PEITC *in vivo*. Significantly higher protein levels of the classic p53 target genes p21, BAX, and PUMA, as well as increased levels of the Bcl2 protein, were detected in the KYSE150 xenograft-tumor-bearing mice treated with PEITC than in the mice in the DMSO control group (Figure 6C); similar phenomena were not observed in the xenograft-tumor-bearing mice with the remaining three esophageal squamous carcinoma cells (Figure 6C). The findings show that PEITC is able to reactivate mutant p53 *in vivo* and inhibit the growth of KYSE150 esophageal squamous carcinoma xenograft tumors in a mouse model. Possible mechanisms of action of PEITC in p53 mutant ESCC (Figure 6D).

## 4 Discussion

Approximately 40% of human tumors carry the p53 missense mutant allele (Schulz-Heddergott and Moll, 2018). Alleles with such mutations produce aberrant proteins that have “dominant negative effects” on wtp53 activity, as well as acquire new oncogenic functions that make the prognosis poor (Dittmer et al., 1993; Muller and Vousden, 2014; Aggarwal et al., 2019). Approximately half of patients with esophageal cancer have p53 mutations. Mutated p53 proteins are abnormally stable and accumulate in the nucleus, and this outcome is a key prerequisite for their GOF (Yue et al., 2017; Schulz-Heddergott and Moll, 2018). p53 overexpression is usually associated with p53 mutations. The meta-analysis results show that p53 overexpression is significantly associated with low 5-year survival in esophageal cancer (Wang et al., 2016). Therefore, we believe mutp53 is a promising cancer-specific drug target, and targeting it with novel small-molecule compounds that can restore its function may open up new strategies for the clinical treatment of a wide variety of patients with p53-mutant cancer, containing those with ESCC. Various small-molecule compounds, peptides, and gene editing tools have been reported to target mutp53 to perform tumor suppressor function, restore the transcriptional activity of mutp53 or induce mutp53 degradation, which are important therapeutic approaches to block or prevent tumor progression (Lambert et al., 2009; Bykov et al., 2016; Schulz-Heddergott and Moll, 2018; Aggarwal et al., 2019; Chen et al., 2021). There is, however, a lack of studies looking at the potential of dietary-related molecules in targeting p53 mutants, especially for esophageal cancer.

PEITC is an isothiocyanate compound present in cruciferous vegetables that has cancer chemopreventive and therapeutic properties. Although PEITC has been shown to selectively deplete p53 mutants owing to the antitumor effects of p53, most studies have focused on its structural mutations, with few studies on exploring these DNA contact mutations. The diversity of p53 mutations compels us to conduct more research to gain a more comprehensive understanding of the effect of PEITC on various cancer types and different p53 mutations.

Herein, we found that PEITC reactivated the p53^R248Q^ mutant hotspot in ESCC cells, restoring wtp53 function and thus exerting antitumor effects. Although PEITC can selectively and preferentially act on ESCC cells expressing mutp53, we also observed that PEITC is able to inhibit proliferation and induce apoptosis in wtp53 ESCC cells, which may be attributed to p53-dependent induction of apoptosis by PEITC in wtp53 cells (Huang et al., 1998). Thus, the decreased viability and increased apoptosis of EC109, shp53-EC109 and shp53-KYSE150 cells caused by PEITC are related to the pathway independent of p53 mutation state, such as p53-independent form of lysosome-dependent programmed cell death and alkaliptosis (Raj et al., 2022), which is consistent with previous studies (Huang et al., 1998; Xiao and Singh, 2002; Aggarwal et al., 2016).

Most studies have concluded that the main feature of p53^R248Q^ is the inability of the DBD to bind to DNA rather than a significant conformational change in DBD, thus classifying it as a DNA contact mutation (Hollstein et al., 1991; Olivier et al., 2010; Soragni et al., 2016). Some researchers believe that p53 mutations are complex and diverse and cannot be classified in absolute terms (Joerger and Fersht, 2008; Ng et al., 2015). Wong et al. demonstrated the existence of structural alterations in R248Q by NMR experiments (Wong et al., 1999) and that the chemical shift and thermal stability of R248Q are similar to those of R249S (a known structural mutant) (Bullock et al., 1997; Bullock et al., 2000); therefore, p53^R248Q^ is also a structural mutant. Consistent with the latter view, our study showed that PEITC can induce conformational changes in p53^R248Q^ protein, restoring it to a “WT-like” conformation. Therefore, we conclude that p53^R248Q^ is both a DNA contact mutation and a structural mutation and cannot be definitively categorized into either class of DNA.

As expected, PEITC restored the transactivating function of p53^R248Q^, consistent with the view that PEITC can reactivate structural or contact mutant p53 (Aggarwal et al., 2019). We observed increased expression of the classical targets of p53. This is consistent with the transcriptional reactivation of p53^R248Q^ in tumor-like organs by the cell-penetrating peptide ReACp53(Soragni et al., 2016). ChIP assays also confirmed that PEITC activated p21 transcription and increased p21 mRNA expression levels in p53^R248Q^ ESCC cells but had no effect on WT p53 ESCC cells. Reactivated p53 behaved similarly to wtp53, inhibiting cell proliferation and promoting cell death (Aggarwal et al., 2016; Soragni et al., 2016; Aggarwal et al., 2019). Consistent with studies on p53^R175^, PEITC induced the expression of several proapoptotic targets in our study, and the expression of the proapoptotic gene Bcl2 was increased. The main reason for the failure of Bcl2 to hedge against PEITC-induced cell death may be that PEITC induces apoptosis in p53-mutant ESCC cells by the caspase pathway, and another reason may be that the relative level of BAX is higher than that of Bcl2, which results in an increases BAX homodimers thereby promoting cell death. Thus, the mechanisms of PEITC-induced cell death are intricate, and studying how genes interact would be interesting. The p53-mediated upregulation of p21 inhibited CyclinB1/CDK1 expression, leading to arrest at G2/M transition (Sperka et al., 2012). In favor of this, we observed that PEITC-treated p53^R248Q^ ESCC cells were arrested at the G2/M phase, while CyclinB1 protein expression was reduced in the cells. These results provide further evidence that PEITC-induced p53 mutant rescue is able to abolish cancer-promoting GOF activity and exerts anti-tumor function.

p73 can respond to DNA damage and other stress signals that activate p53, thereby inhibiting cell proliferation or inducing apoptosis (Riley et al., 2008; Lunghi et al., 2009). Although one of the main GOF activities of mutant p53 is to antagonize p63/p73 and thereby inhibit its tumor suppressor function (Zhang J. et al., 2019), the underlying molecular mechanisms have not been elucidated. Certain kinds of p53 mutants (e.g., p53^R175H^ and p53^R273H^) were shown to exert GOF activity through direct interactions with p63 or p73 in earlier studies (Gaiddon et al., 2001; Stindt et al., 2015). We show that restoration of the p53^R248Q^ mutant by PEITC suppresses its ability to bind to p73. This is consistent with earlier findings. Recent studies have shown that mutant p53 represses the p63/p73-regulated transcriptional activities through the Notch1 pathway (Zhang J. et al., 2019). These data suggest an intricate association between mutp53 GOF and p73. Surprisingly, both the mRNA and protein levels of p73 were downregulated in our study, which we speculate may be a consequence of the recovery of the p53^R248Q^ mutant. This is inconsistent with the findings of Monika Aggarwal et al. for the p53^P223L^ mutant and warrants further investigation (Aggarwal et al., 2019). In conclusion, these findings indicate that PEITC-induced recovery of mutant p53 also suppresses its GOF activity. It is extremely important to investigate the effects of PEITC on other GOF activities of the recovered mutant p53 in further detail (Liu et al., 2014; Aggarwal et al., 2016; Aggarwal et al., 2019).

Experiments with an *in vivo* transplantation tumor model also confirmed PEITC-mediated antitumor responses via reactivation of mutp53. Although the p53 antibody used for immunohistochemistry in this study could not distinguish a wtp53 and mutp53, several researches have indicated that the p53 protein detected by immunohistochemistry represents most of mutant p53 proteins (Feng et al., 2002; Ishiguro et al., 2016; Kokkinos et al., 2021). Combining *in vitro* and *in vivo* experiments, cancer cells were found to become less aggressive when mutp53 was knocked down, suggesting that mutp53 facilitates cancer cell survival and proliferation. As for safety, previous studies have shown that PEITC has genotoxicity *in vitro* experiments, but not *in vivo* experiments (Kassie and Knasmuller, 2000; Suzuki et al., 2017). PEITC genotoxicity will be studied in future subsequent experiments. In this study, it was observed that PEITC had selective antitumor effect but no significant effect on the body weight of mice, no accidental death of mice, and the cell morphology of the important organs of mice in each group was good without obvious injury, necrosis and apoptosis (Supplementary Figure S6). Therefore, we believed that PEITC had selective cytotoxic effect with low inherent toxicity and good safety. Overall, the study of the role of PEITC in p53^R248Q^ ESCC has led to a deeper understanding of the function of dietary compounds and p53 mutants, combined with the role of PEITC in breast cancer (SK-BR-3 (p53^R175H^), AU565 (p53^R175H^)) and prostate cancer (LAPC-4 (p53^R175H^) and VCaP (p53^R248W^)) (Aggarwal et al., 2016; Aggarwal et al., 2019), it is plausible that PEITC could be used in “basket trials” for cancers with selective p53 mutations, making it possible to develop new strategies for treating most cancers in the future.

p53 gene mutations occur at different stages of esophageal cancer tumorigenesis (Agrawal et al., 2012), suggesting that p53 mutants could be a therapeutic target for this cancer. In theory, pharmacological reactivation of mutant p53 is a good option for targeting cancer cells for therapy. However, to date, none of the “reactivators” or “eliminators” have been successfully applied in the clinic, highlighting the importance of finding pharmacological alternatives that are more selective and less toxic. PEITC may be a promising candidate, demonstrating excellent anticancer efficacy either alone or in combination with chemotherapeutic agents. Therefore, it is expected to open up new treatment modalities for cancer patients with high frequency of p53 mutation, including ESCC. However, the study of p53 mutation types in ESCC was not comprehensive enough to research the role of PEITC in p53 structural mutations, DNA contact mutations, and p53-null ESCC cells. More basic researches and clinical studies are needed to further explore this.

## Data Availability

The raw data supporting the conclusions of this article will be made available by the authors, without undue reservation.

## Abbreviations

mutp53, mutant p53; PEITC, phenethyl isothiocyanate; ESCC, esophageal squamous cell carcinoma; wtp53, wild-type p53; ITCs, isothiocyanates; FBS, fetal bovine serum; DMSO, dimethyl sulfoxide; PI, propidium iodide; RT-qPCR, reverse transcription-quantitative PCR; CoIP, coimmunoprecipitation; ChIP, chromatin immunoprecipitation; HE, hematoxylin and eosin; IHC, immunohistochemistry; DAB, diaminobenzidine; GOF, gain of function; DBD, DNA binding domain.
