# Supplementary material for: Reactivation of mutant p53 in esophageal squamous cell carcinoma by isothiocyanate inhibits tumor growth
Source: Front Pharmacol. 2023 Apr 24;14:1141420. doi: 10.3389/fphar.2023.1141420 (PMC10164965; doi:10.3389/fphar.2023.1141420)
Supplement: Supplementary file 1 [file DataSheet1.DOCX]

Supplementary Material

# Supplementary Table

Table 1 The primers for quantitative PCR

| Gene | Forward primer | Reverse primer |
| --- | --- | --- |
| GAPDH | GGAGCGAGATCCCTCCAAAAT | GGCTGTTGTCATACTTCTCATGG |
| p21 | TGTCCGTCAGAACCCATGC | AAAGTCGAAGTTCCATCGCTC |
| BAX | CCCGAGAGGTCTTTTTCCGAG | CCAGCCCATGATGGTTCTGAT |
| Bcl2 | TTGCCAGCCGGAACCTATG | CGAAGGCGACCAGCAATGATA |
| PUMA | GACCTCAACGCACAGTACGAG | AGGAGTCCCATGATGAGATTGT |
| MDM2 | TCGTCGGGTGAGGGTACTG | AACCACTTCTTGGAACCAGGT |
| p73 | ATGGAGACGAGGACACGTACTACC | GCTGCTGCTGCTGCCGATAG |

# Supplementary Figures


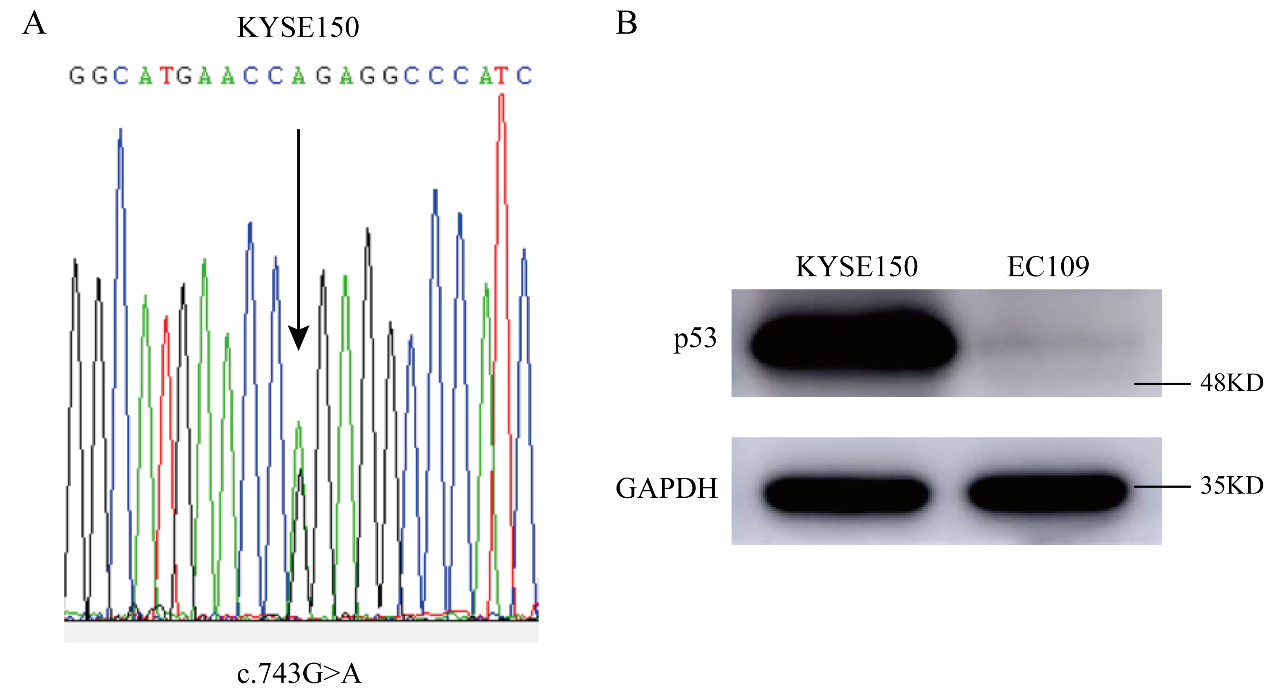


**Supplementary Figure 1.** Mutation status and protein expression of p53 gene. (A) Mutation peak map of p53 gene in KYSE150 cells. Exon 7 missense mutation c.743G>A, black arrow represents the mutation site. (B) Expression levels of p53 protein in KYSE150 and EC109 cells.


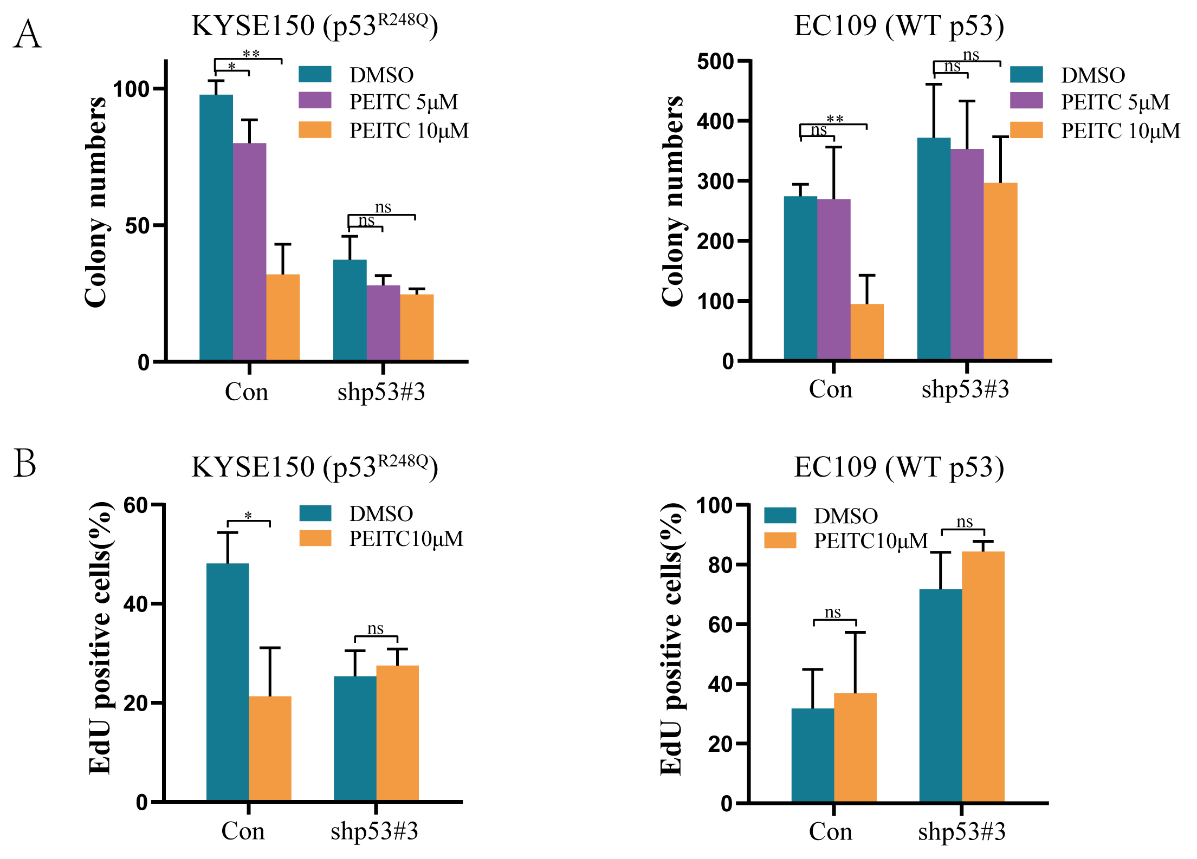


Supplementary Figure 2 Effect of PEITC on the proliferation of ESCC cells with different p53 gene status. (A) Statistical histogram of clone formation assay. (B) Statistical histogram of EdU incorporation assay.


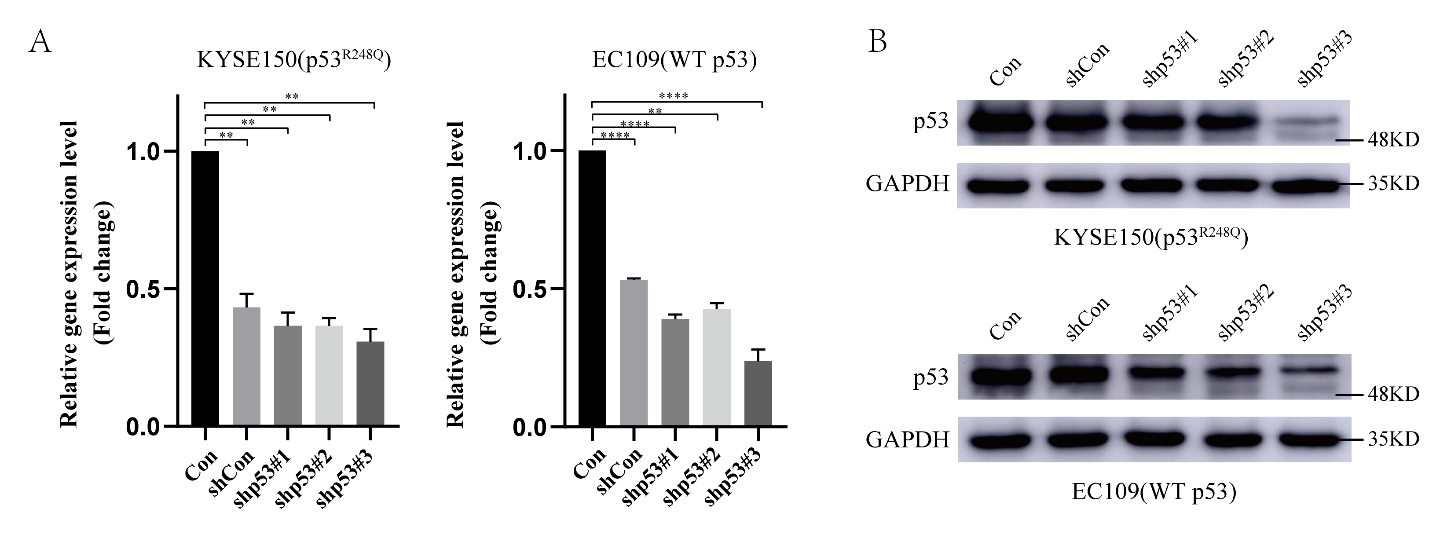


Supplementary Figure 3 p53 knockdown efficiency in KYSE150 and EC109 cells. (A) Expression levels of p53 mRNA in cells transfected with lentivirus. (B) Expression levels of p53 protein in cells transfected with lentivirus.


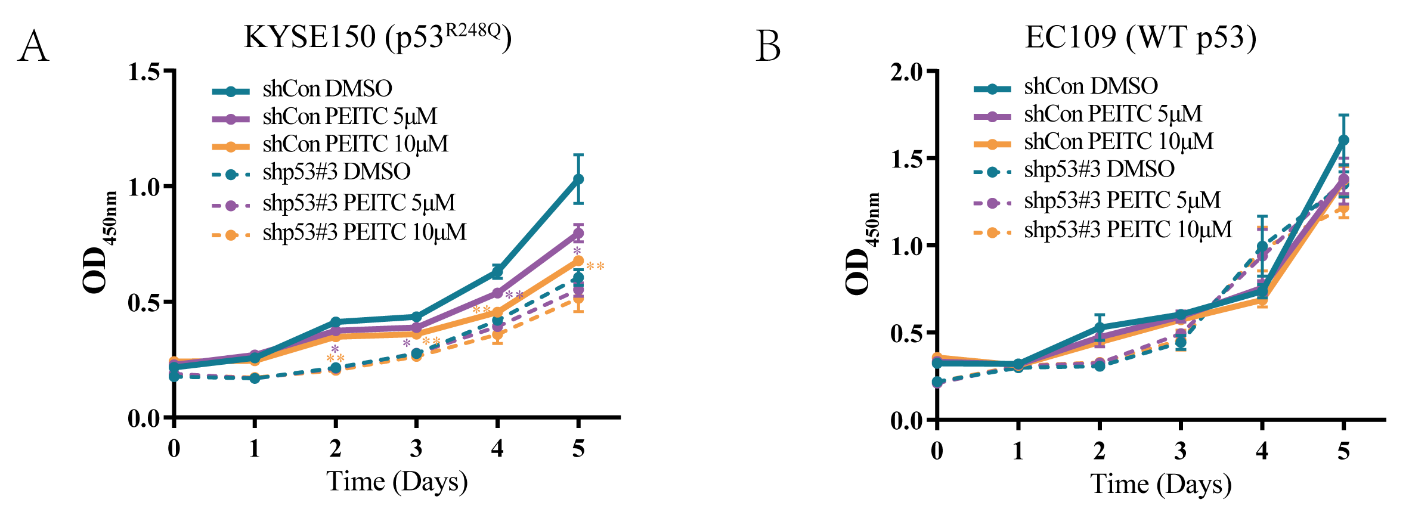


Supplementary Figure 4 PEITC inhibits proliferation in a p53^R248Q^-dependent manner. (A, B) Proliferation of cells was determined using the CCK-8 assay.


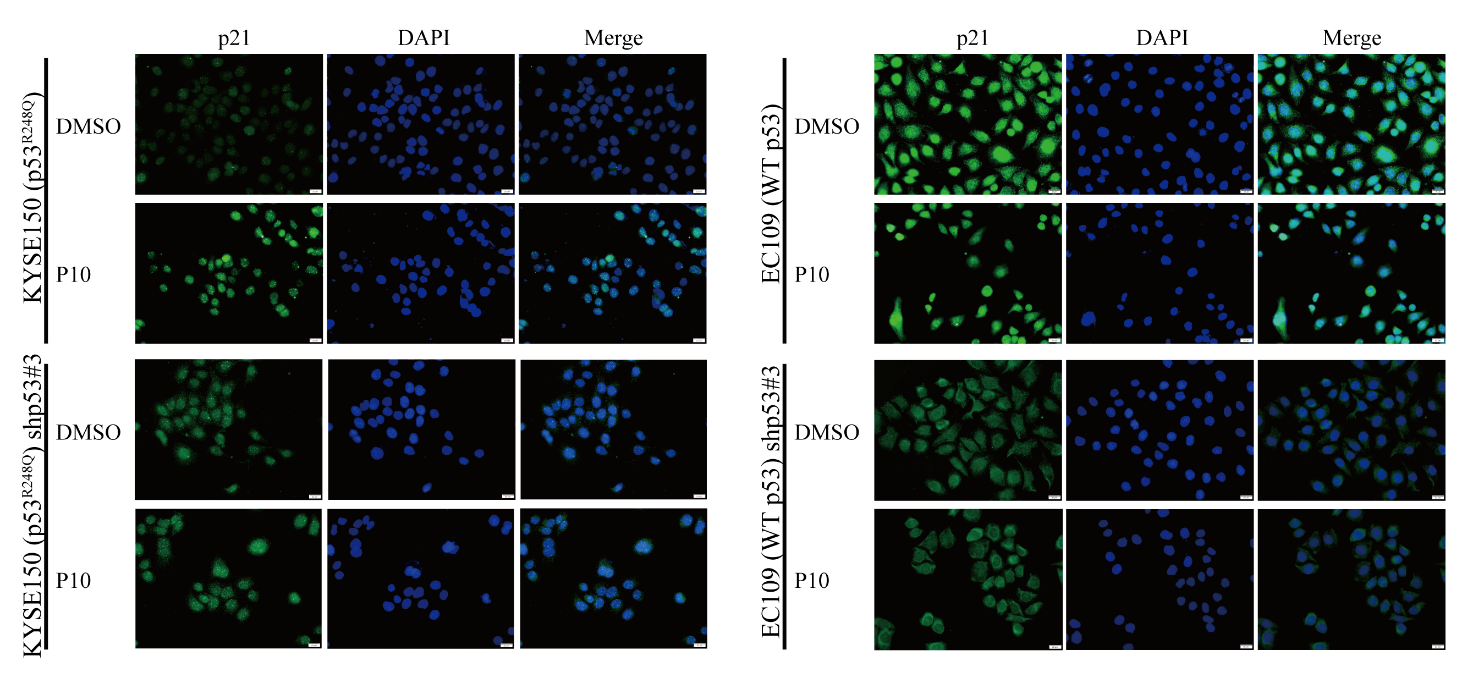


Supplementary Figure 5 Immunofluorescence staining for p21 protein in KYSE150 and EC109 cell lines.


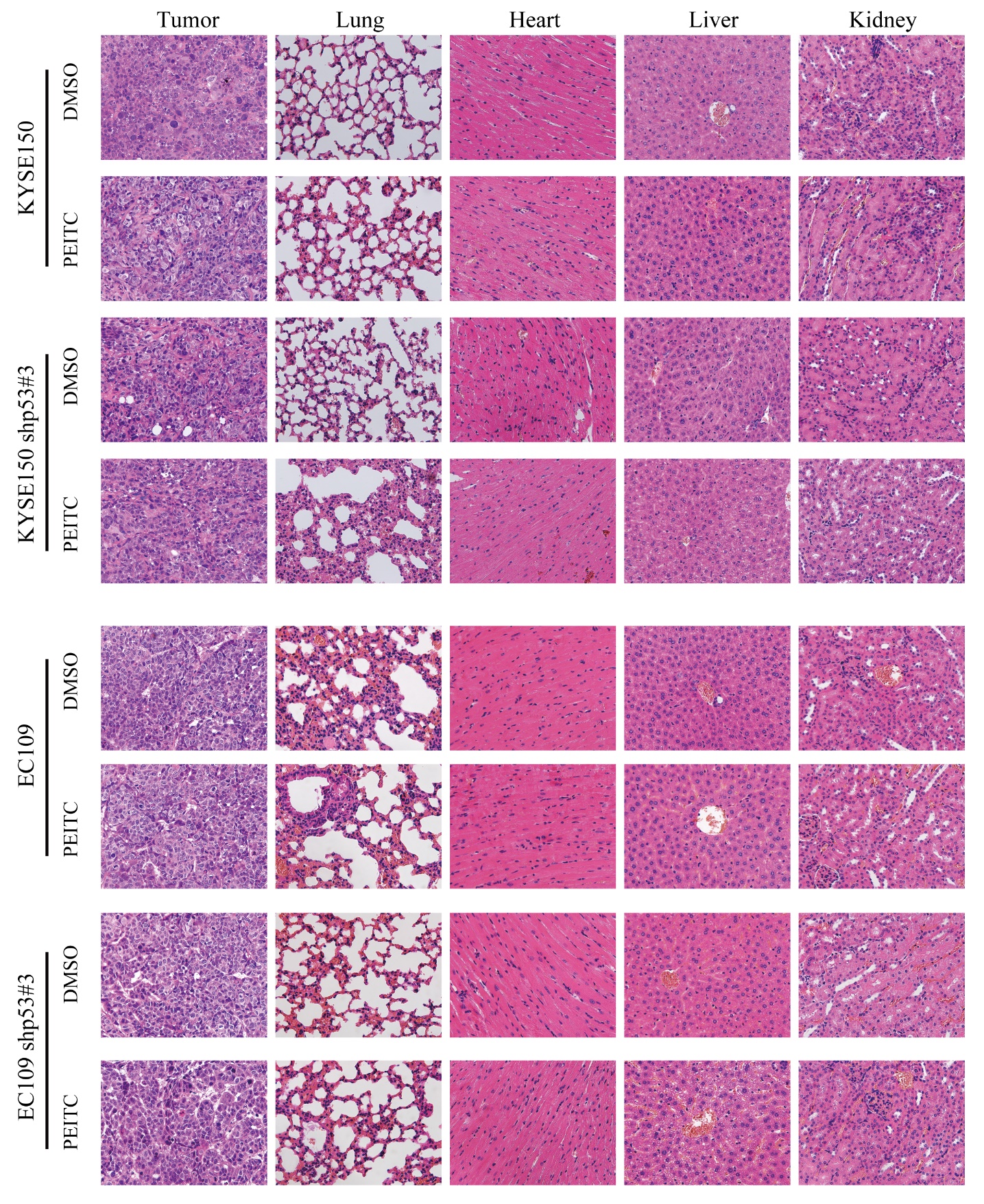


Supplementary Figure 6 PEITC is not toxic to nude mouse organs. Representative images of HE staining of vital organs in nude mice.
